# Recommendations for sex/gender neuroimaging research: key principles and implications for research design, analysis, and interpretation

**DOI:** 10.3389/fnhum.2014.00650

**Published:** 2014-08-28

**Authors:** Gina Rippon, Rebecca Jordan-Young, Anelis Kaiser, Cordelia Fine

**Affiliations:** ^1^Aston Brain Centre, School of Life and Health Sciences (Psychology), Aston UniversityBirmingham, West Midlands, UK; ^2^Department of Women’s, Gender and Sexuality Studies, Barnard College, Columbia University in the City of New YorkNew York, NY, USA; ^3^Department of Social Psychology, Institute of Psychology, University of BernBern, Switzerland; ^4^Melbourne School of Psychological Sciences, Melbourne Business School, and Centre for Ethical Leadership, University of MelbourneCarlton, VIC, Australia

**Keywords:** brain imaging, sex differences, sex similarities, gender, stereotypes, essentialism, plasticity

## Abstract

Neuroimaging (NI) technologies are having increasing impact in the study of complex cognitive and social processes. In this emerging field of social cognitive neuroscience, a central goal should be to increase the understanding of the interaction between the neurobiology of the individual and the environment in which humans develop and function. The study of sex/gender is often a focus for NI research, and may be motivated by a desire to better understand general developmental principles, mental health problems that show female-male disparities, and gendered differences in society. In order to ensure the maximum possible contribution of NI research to these goals, we draw attention to four key principles—overlap, mosaicism, contingency and entanglement—that have emerged from sex/gender research and that should inform NI research design, analysis and interpretation. We discuss the implications of these principles in the form of constructive guidelines and suggestions for researchers, editors, reviewers and science communicators.

## Introduction

Over the past few decades, psychologists have documented a tendency for lay-people to hold “essentialist” beliefs about social categories, including gender (for summary, see Haslam and Whelan, 2008). Essentialist thinking about social categories involves two important dimensions (Rothbart and Taylor, 1992; Haslam et al., 2000). Essentialized social categories are seen as “natural kinds”, being natural, fixed, invariant across time and place, and discrete (that is, with a sharply defined category boundary). In addition, essentialized social categories are “reified”, being seen as “inductively potent, homogenous, identity-determining, and grounded in deep, inherent properties” (Haslam and Whelan, 2008, p. 1299).

Gender is a strongly essentialized category, particularly in the degree to which it is seen as a natural kind (Haslam et al., 2000), with interpersonal differences spontaneously interpreted through a gendered lens (Prentice and Miller, 2006). 3G-sex (that is, the genetic, gonadal, and genital endowment, of an individual (Joel, 2011)) is indeed highly—although not completely—internally consistent, discrete and invariant across time and place and thus much more of a “natural kind”. Yet decades of gender scholarship have undermined the traditional essentialist view of the behavioral manifestations of masculinity and femininity, and their neural correlates, which are of interest to neuroscientists (Schmitz and Höppner, 2014).

The key principles from gender scholarship of overlap, mosaicism, contingency, and entanglement, reviewed in the following sections, offer a serious challenge to essentialist notions of sex/gender^1^ as fixed, invariant, and highly informative. This is an important message for neuroscientists because, unless they have specific expertise or knowledge in gender scholarship, they too are laypeople with respect to gender research, and may also be susceptible to gender essentialist thinking. Indeed, sex/gender NI^2^ research currently often appears to proceed as if a simple essentialist view of the sexes were correct: that is, as if sexes clustered distinctively and consistently at opposite ends of a single gender continuum, due to distinctive female vs. male brain circuitry, largely fixed by a sexually-differentiated genetic blueprint. Data on the sex of participants are ubiquitously collected and available; the two sexes may be routinely compared with only positive findings reported (Maccoby and Jacklin, 1974; Hines, 2004); and the emphasis on difference is institutionalized in databases that allow only searches for sex/gender differences, not similarities (Kaiser et al., 2009).

The all but ubiquitous group categorization on the basis of biological sex seems to suggest the implicit assumption that a person’s biological sex is a good proxy for gendered behavior and that therefore categorizing a sample on the basis of sex will yield distinct “feminine” vs. “masculine” profiles. The small sample sizes common in fMRI investigations reporting female/male differences (Fine, 2013a) suggests the implicit assumption that female vs. male brain functioning is so distinct that true effects can be identified with small numbers of participants. Conversely, with large sample sizes (seen mostly in structural comparisons), the publication of statistically significant effects suggests the implicit assumption that they are also of theoretical and functional significance. The readiness with which researchers draw on gender stereotypes in making reverse inferences (Bluhm, 2013b; Fine, 2013a) suggests an implicit assumption of distinctive female vs. male brains giving rise to “feminine” and “masculine” behavior, respectively. Finally, the common use of single “snapshot” female/male comparisons (Schmitz, 2002; Fine, 2013a) is in keeping with the implicit assumption of gendered behavior and female and male brains as fixed and non-contingent, meaning that such an approach promises to yield “the” neural difference between the sexes for a particular gendered behavior.

Thus, our goal in this article is to draw attention to the four key principles of overlap, mosaicism, contingency and entanglement that have emerged from sex/gender research, and discuss how they should inform NI research design, analysis and interpretation.

## Principles from sex/gender scholarship

### Overlap

Studies examining sex/gender typically categorize participants as female or male and apply statistical procedures of comparison. Sex/gender differences in social behavior and cognitive skills are, if found, far less profound than those portrayed by common stereotypes. As Hyde (2005) found in her now classic review of 46 meta-analytic studies of sex differences, scores obtained from groups of females and males substantially overlap on the majority of social, cognitive, and personality variables. Of 124 effect sizes (Cohen, 1988)^3^ reviewed, 30% were between (+/−) 0 and 0.1 (e.g., negotiator competitiveness, reading comprehension, vocabulary, interpersonal leadership style, happiness), while 48% were between (+/−) 0.11 and 0.35 (e.g., facial expression processing in children, justice vs. care orientation in moral reasoning, arousal to sexual stimuli, spatial visualization, democratic vs. autocratic leadership styles). There is non-trivial overlap even on “feminine” and “masculine” characteristics such as physical aggression (*d* ranges from 0.33 to 0.84), tender-mindedness (*d* = −0.91), and mental rotation (*d* ranges from 0.56 to 0.73). More recent reviews have also emphasized the extent of this overlap (Miller and Halpern, 2013; Hyde, 2014).

There are more significant differences between women and men in other categories of behavior, such as choice of occupations and hobbies (Lippa, 1991). However, regardless of how one wishes to characterize the data (that is, as demonstrating that females and males are “different” or “similar”), or the functional significance of differences of a particular size (considerable or trivial), the important point for NI researchers is that the distributions of social cognitive variables typically of interest in research are likely to be highly overlapping between the sexes, and this has implications for research design. It has also been argued that many small differences may “add up” to very significant differences overall (Del Giudice et al., 2012; Cahill, 2014, although for critique of the latter, see Stewart-Williams and Thomas, 2013; Hyde, 2014). However, not only does this argument overlook the “mosaic” structure of sex/gender (discussed in the next section) but, additionally, NI researchers will generally be interested in isolating just one or two behavioral variables.

Overlap in behavioral phenotype does not necessarily imply overlap in cortical structural and functional phenotype, since potentially the same behavioral ends may be reached via different neural means—an important point when it comes to interpretation of group differences in neural characteristics (Fine, 2010b; Hoffman, 2012). Indeed, it has been noted in non-human animals that one average difference between the sexes in a brain characteristic may compensate for another, giving rise to behavioral similarity (De Vries, 2004). However, it nonetheless appears to be the case that establishing non-ephemeral sex/gender differences in cortical structures and functions has proved difficult. One commonly cited difference, supported by several meta-analyses and reviews, is that absolute brain volume is greater in men than in women (Lenroot and Giedd, 2010; Sacher et al., 2013) even when body size is controlled for (Cosgrove et al., 2007), although, as with psychological characteristics, the distributions overlap considerably. The significance of this is that, once volume differences are controlled for, many previously reported *regional* differences in specific structures disappear (e.g., Leonard et al., 2008). For instance, the claim that callosal size is greater in males is not supported when there is careful matching between the sexes in brain-size (Bishop and Wahlsten, 1997; Jäncke et al., 1997; Luders et al., 2014). However, this may not invariably be the case, with clusters of regional female/male differences in gray matter found to persist even in female and male participants matched for brain size (Luders et al., 2009), consistent with some previous findings (e.g., Good et al., 2001; Luders et al., 2006) but not all (Lüders et al., 2002). In addition, Giedd et al. (2012) note that the non-linear scaling relationship between brain size and brain proportions affects white to gray matter ratios, which could account for female/male differences in this measure.

It is also important to note that it has proved difficult to replicate well-accepted reports of sex/gender differences in *functional* organization of brain regions underpinning specific cognitive skills. A salutary example of this is the long-standing hypothesis that the male brain is more lateralized for language processing. A high-impact report that partially supported this hypothesis (Shaywitz et al., 1995, see Kaiser et al., 2009) was subsequently shown to be spurious in two meta-analytic studies (Sommer et al., 2004, 2008).

The substantive point here is not to argue that there are *no* structural or functional brain differences between the sexes, but to draw attention to the fact that neural characteristics are not so distinctly different in the sexes that reliable differences are easily identified. These data make it clear that dimorphism, the existence of two distinct forms, is not an accurate way to characterize sex/gender differences in neural phenotype.

### Mosaicism

Developments in understanding of the structure of gender (that is, the traits, roles, behaviors, attitudes, and so on, associated with femininity and masculinity) have challenged the earlier assumption that the sexes cluster distinctively and consistently at opposite ends of a single gender continuum (Terman and Miles, 1936) or can be located on two discrete “feminine” and “masculine” dimensions (Bem, 1974). Because different feminine and masculine characteristics are only weakly inter-correlated, if at all, gender is now understood to be multi-factorial, rather than one-or two-dimensional (Spence, 1993). Similarly, Carothers and Reis (Carothers and Reis, 2013; Reis and Carothers, 2014), applying taxometric methods to analyze the latent structure of gender, have recently concluded that females’ and males’ psychological attributes mostly differ in ways that are continuous rather than categorical.

Similarly in neuroscience, the phenomenon of brain mosaicism has been recognized for decades (Witelson, 1991; Cahill, 2006; McCarthy and Arnold, 2011, see also Joel, 2011). That is, an individual does not have a uniformly “female” or “male” brain, but the “male” form (as statistically defined) in some areas and the “female” form in others, and in ways that differ across individuals. (Nor is this necessarily static, with animal research indicating that even brief experiences such as stress exposure can change brain characteristics from the “female” to the “male” form, and vice versa; see Joel, 2011).^4^ Thus, having a region in (say) the corpus callosum where a structural or functional characteristic has been shown to be statistically more characteristic of females is not a good predictor for whether the same individual brain will also have a region in (say) the amygdala that is associated with females. An implication of this mosaicism is that specific brain areas that are labeled as having a “female” or “male” phenotype can only be detected through group-level statistical comparisons. In other words, just as individuals are not comprehensively feminine or masculine in traits, roles attitudes, etc., so too is it not possible for an individual to have a “single-sex” brain.

Mosaicism of gendered behavior and brains is a critically important point, because it conflicts with the more (although not absolutely) categorical nature of biological sex, in which female/male differences in sex chromosomes, gonads and genitals are roughly dimorphic and highly interrelated, such that individuals mostly have a unitary “male” or “female” phenotype. As Joel (2012) has put the issue, “Using 3G-sex (genetic-gonadal-genitals) as a model to understand sex differences in other domains (e.g., brain, behavior) leads to the erroneous assumption that sex differences in these other domains are also highly dimorphic and highly consistent” (p. 1). Even where mosaicism is acknowledged, the evidence may be undermined by common terminology such as “female or male phenotype” (for describing global brain structure or psychology) or “sex dimorphism” (Jordan-Young, 2014).

### Contingency

Gendered behavior arises out of a dauntingly complex, reciprocally influencing interaction of multi-level factors, including structural-level factors (e.g., prevailing cultural gender norms, policies and inequalities), social-level factors (e.g., social status, role, social context, interpersonal dynamics) as well as individual-level factors such as biological characteristics (see “entanglement” principle in the following section), gender identity, gendered traits, attitudes, self-concepts, experiences, and skills. A few illustrative examples, which depart from the more “intuitive” conception of sex/gender differences as emerging from a causal pathway that runs from genes to hormone to brain to behavior to social structure, may be useful.

At the group level, women’s expression of “masculine” personality traits (such as assertiveness) appears to be responsive to cultural shifts in social status and role (Twenge, 1997, 2001), while in the shorter term, gendered behavior is flexibly responsive to social context and experience. For example, a meta-analysis conducted by Ickes et al. (2000) found that a moderate female advantage in empathic accuracy was only observed if participants were also asked to make self-ratings of their accuracy (hypothesized to preferentially enhance women’s motivation to perform well). Another well-known example of social contextual effects on gendered behavior is the “stereotype threat” phenomenon whereby, for instance, female mathematical performance is diminished when tests are presented in a way that makes salient the stereotype that females are poor at mathematics (Nguyen and Ryan, 2008; Walton and Spencer, 2009), although we acknowledge the more sceptical conclusion regarding the size, robustness, and generality of the stereotype threat effect from the meta-analysis by Stoet and Geary, 2012. As a third example, the average male advantage in mental rotation is diminished by altering how the task is framed (e.g., Moè, 2009). Moreover, the beneficial effects of training, including video-gaming, points to the contribution of gendered experience to this skill (Feng et al., 2007). (For numerous additional examples of stereotype threat effects on sex/gender differences, see Fine, 2010a).

From this brief discussion it should therefore not be surprising that, in contrast with the near complete consistency of genetic, gonadal and genital differences between the sexes, female/male differences in *behavior* are variable across time, place, social or ethnic group, and social situation. Indeed, intersectionality—the principle that important social identities like gender, ethnicity, and social class “mutually constitute, reinforce, and naturalize one another” (Crenshaw, 1991, p. 302)—is an important tenet of gender scholarship (Crenshaw, 1991; Shields, 2008). For example, as reviewed in Hyde (2014), female/male differences in mathematics in the USA have not only decreased over time but also vary or even reverse according to ethnic group. A review of differences in math achievement in 69 nations by Else-Quest et al. (2010) revealed that gender differences were not only very small, but highly variable, with effect sizes ranging from −0.42 (a moderate difference favoring females) to 0.40 (a moderate difference favoring males); socio-cultural factors such as women’s parliamentary representation, equity in school enrolment, and women’s share of research jobs were significant predictors of gender gaps in math achievement. As with cognitive skills, female/male differences in personality (e.g., neuroticism/anxiety) or well-being (e.g., self-esteem) that are seen in one country or ethnic group are not necessarily observed in others (Costa et al., 2001, reviewed in Hyde, 2014).

### Entanglement

As indicated above, there is considerable evidence that average female/male differences can be modified, neutralized, or even reversed by specific context, for example the manipulation of the salience of such differences, or by chronic structural factors in the environment, such as national wealth or gender equity (reviewed in Miller and Halpern, 2013; Hyde, 2014). Clearly, this will be reflected in the neural substrates of such behavior, which therefore cannot be universal or fixed (see Fine, 2013b). This type of finding is in keeping with the rejection of early models of the relationship between brain and behavior in the study of sex/gender. These were based on a fairly simple, almost unidirectional concept of “hard-wiring”, in which brain characteristics were conceived as being predetermined by the organizational effects of genetically-programmed prenatal hormonal influences (Phoenix et al., 1959). Here, each individual is endowed with a “female” or “male” brain that gives rise to feminine and masculine behavior, respectively; a neural substrate that social factors merely influence. This assumption of distinctive female vs. male brain circuitry, largely fixed by a sexually-differentiated genetic blueprint, is now clearly challenged by changed models of neurodevelopment and wide-spread consensus of on-going interactive and reciprocal influences of biology and environment in brain structure and function (Li, 2003; Lickliter and Honeycutt, 2003; van Anders and Watson, 2006; Hausmann et al., 2009; McCarthy and Arnold, 2011; Miller and Halpern, 2013). As NI research itself has been instrumental in demonstrating, such interactions leave neural traces. A recent review by May (2011) summarizes the evidence that new events, environmental changes and skill learning can alter brain function and the underlying neuroanatomic circuitry throughout our lives. Such changes could be brought about by, for example, normal learning experiences such as learning a language (Stein et al., 2012) or specific training activities such as taxi-driving or juggling (Maguire et al., 2000; Draganski et al., 2004; Chang, 2014). Other research demonstrates brain characteristics that vary as a function of socio-economic status (Hackman and Farah, 2009; Noble et al., 2012) or even subjective or perceived socio-economic status (Gianaros et al., 2007). Despite the key role played by NI research in the emergent concept of the permanently plastic brain, only a few NI studies have demonstrated how neuronal plasticity has been related to sex/gender. Wraga et al. (2006), using a direct comparison of task-related positive and negative stereotype priming, showed that the neural correlates of performance of the same task reflected this priming, demonstrating short-term plasticity of neural function. Longer-term functional and structural plasticity was indicated in another within-sex study investigating the neural effects in adolescent girls of 3 months of training with the visuo-spatial problem solving computer game Tetris (Haier et al., 2009).

This dynamic and interactive conception of brain development means that biological sex and the social phenomenon of gender are “entangled” (Fausto-Sterling, 2000). That is, as a categorization linked to social difference and inequality, an individual’s biological sex systematically affects their psychological, physical, and material experiences (Cheslack-Postava and Jordan-Young, 2012; Springer et al., 2012). For example, because gender is an important organizing principle for social life, giving rise to intensive gender socialization, including self-socialization processes (e.g., Bussey and Bandura, 1999; Martin and Ruble, 2004; Leaper and Friedman, 2007; Tobin et al., 2010), both formal training (e.g., school and vocational instruction) and daily experiences (e.g., sports involvement, hobbies, games, poverty, and harassment) are, at the group level, different for females and males. It will be critical for NI work investigating hormone-brain relations to take into account important insights into entanglement from social neuroendocrinology. Contemporary models identify hormones such as testosterone as key mediators of behavioral plasticity, with animal research indicating both genomic and non-genomic mechanisms involving both long-term structural reorganization and short-term modulation of sensitivity of neural circuitry (Adkins-Regan, 2005; Oliveira, 2009). This enables animals to be flexibly responsive to social situations that, in humans, incorporate gendered norms with respect to social phenomena such as competition, sexuality, and nurturance (van Anders, 2013). For example, it has been shown that fatherhood can reduce testosterone levels in males and that this effect varies with the extent of paternal care and physical contact with offspring (Gettler et al., 2011). Furthermore, a comparison of two neighboring cultural groups in Tanzania found lower testosterone levels among fathers from the population in which paternal care was the cultural norm compared with fathers from the group in which paternal care was typically absent (Muller et al., 2009). Entanglement thus refers to the fact that the social phenomenon of gender is literally incorporated, shaping the brain and endocrine system (Fausto-Sterling, 2000), becoming “part of our cerebral biology” (Kaiser et al., 2009, p. 57).

### Key principles: summary

**Figure 1:**
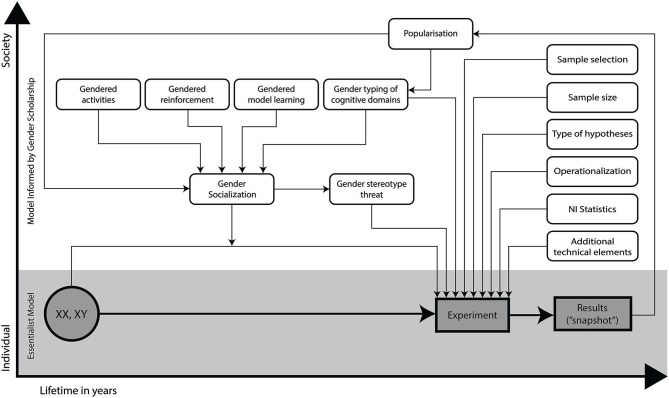
**Comparison of “Essentialist” vs. “Social Context” models of experimental design in sex/gender research**. (Shaded section): the essentialist model that is often implicit in NI sex/gender research: female-male differences appear to be directly traceable to initial genetic differences between female and male individuals. (Unshaded section): the social context model where social context variables interact with individual biologies (contingency) and create feedback loops with research design and practices (entanglement): results of particular studies are understood as contingent and entangled “snapshots”.

The issues identified above indicate that, for NI researchers wishing to examine sex/gender variables in studies of the human brain, there are key factors which need to be taken into consideration in the design, analysis, and interpretation of research in this category. As illustrated in Figure 1, there will need to be adjustments made to the assumptions underlying current typical research practices. As will by now be clear from the discussion of the key principles of sex/gender scholarship, gender essentialist assumptions are inappropriate, and the experimental context complex and contingent. Any one sample will consist of individuals with an intricate mosaic of gendered attributes, the distributions for many of which will be largely overlapping and may not differ at the group level in that particular sample. Similarly, the individuals in the sample will not have “female” or “male” brains as such, but a mosaic of “feminine” and “masculine” characteristics. Whatever female/male behavioral and therefore brain differences are observed in that particular sample are contingent on both chronic and short-term factors such as social group (such as social class, ethnicity), place, historical period, and social context and therefore cannot be assumed a priori to be generalizable to other populations or even situations (such as the same task performed in a different social context). Each individual’s behavioral and neural phenotype at the moment of experimentation is the dynamic product of a complex developmental process involving reciprocally influential interactions between genes, brain, social experience, and cultural context. Simpler, implicitly essentialist models (see lower, shaded portion of Figure 1) will need to be replaced by more complex multivariate models which acknowledge the interactive contribution of many additional sociocultural factors (see upper portion of Figure 1).

**Figure 2:**
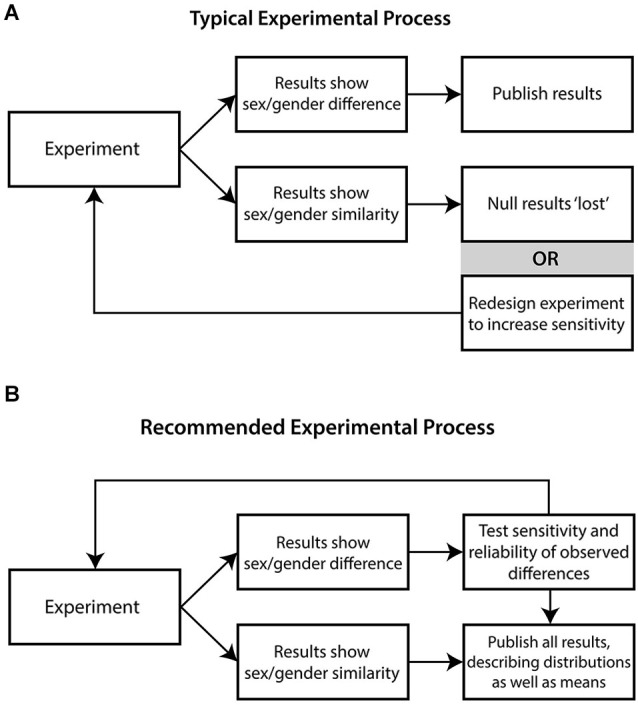
**Comparison of “typical” vs. “recommended” processes in NI research**. **(A)** Typical experimental process in NI research on sex/gender is oriented towards identifying differences. Biological sex is considered primary; two sexes are routinely compared, and findings of “no difference” are often lost (though this may also stimulate redesign of study to better detect difference). **(B)** The recommended experimental process proceeds from the principle of overlap; when differences are observed, researchers attempt to discern the reliability and sensitivity of these observations to social and experimental context. Reports place equal emphasis on findings of sex/gender difference and similarity, with emphasis on distributions.

So what strategies do these key principles of sex/gender scholarship imply for NI sex/gender research design, methodology, and interpretation? We now outline some of the key implications and recommendations for research design, data analysis, and interpretation, which we hope will result in changes from standard practices (as illustrated in Figure 2A) to greater acknowledgment of gender similarities as well as differences, follow-up replication studies, and assessment of effect stabilities where differences are found (see Figure 2B). We conclude with a few comments concerning how these issues relate to ongoing discussions regarding discipline-wide practices.

## Recommendations

### Research design

#### Sample size

Ultimately, sex/gender social and cognitive neuroscience is concerned with the relationship between behavior and the brain, and it is therefore critical that researchers be aware that the key principle of overlap means that participants divided on the basis of biological sex cannot be assumed to have neatly distinct behavioral or cortical structural or functional profiles. Where there is considerable overlap in distribution of scores between a grouping factor (e.g., sex) and the dependent variable of interest, the magnitude of any difference, or effect size (Cohen, 1988) will be very small. Research designed to measure such a difference will obviously need an adequately large sample size to reliably and consistently identify such differences. Small sample size and associated reduced statistical power has been identified as a central problem in NI research (Carp, 2012; Button et al., 2013), as well as in sex/gender fMRI studies (Kaiser, 2010; Fine, 2013a). This clearly raises a concern regarding the high probability of false-negative findings. However, the low statistical power of many studies also validates considerable concern that many reported statistically significant findings are “false positive”. False-positive errors are arguably the most costly errors in science (Simmons et al., 2011), and can be remarkably persistent despite documented null findings (Fidler, 2011; Fine, 2013a). Although, in theory, the probability of false positive errors should remain the same regardless of sample size, as Fine and Fidler (2014) have noted, a combination of publication bias, data noise, large intersubject variability, and considerable scope for researcher discretion about the construction of dependent variables may mean that, in practice, this is not the case. The difficulty, to date, of establishing reliable, non-controversial sex differences in the brain becomes less surprising in light of the key sex/gender principles discussed here and indicates that studies with small sample sizes will lack adequate statistical power and produce unreliable findings.

#### Independent and dependent variables

The evidence that gendered characteristics are often overlapping and multi-dimensional indicates the usefulness of a dimensional trait-based, rather than categorical sex-based, approach to research (Jordan-Young and Rumiati, 2012). Although in psychology the experimental registration of sex/gender in a multi-parametric way is in its infancy, attempts are being made to trace the many different facets of what is an “enormous conglomeration of socialized, behavioral, cognitive, and culturally embedded biomarkers” (Kaiser, 2014). To give some examples, relevant and multiple information about sex/gender can be assessed through the utilization of questionnaires assessing gendered personality dimensions (Personal Attributes Questionnaire, PAQ; Spence and Helmreich, 1978), gender attitudes (Ambivalent Sexism Inventory, ASI; Glick and Fiske, 1996), self-attributed gender norms (Conformity To Masculine Norms Inventory, Mahalik et al., 2003, Conformity To Feminine Norms Inventory, CMFI; Mahalik et al., 2005), specific aspects of gender socialization (The Child Gender Socialization Scale, Blakemore and Hill, 2008), gender identity (Joel et al., 2013) and others (for reviews, see Smiler and Epstein, 2010; Moradi and Parent, 2013). A multi-parametric registration of sex/gender combines multiple binary classifications in various ways, similar to the mosaic-approach of Joel (2011). Most importantly, it promises to emphasize the multi-dimensionality of the factor sex/gender which is usually only measured by checking the F or M box (see Figure 1). In this way, specific sex/gender related information about gendered experiences, gendered socialization, gendered behavior, gendered cognition could be collected. With the emergent availability of large neuroimaging (NI) datasets, much more subtle interrogation of these data would be possible if the demographic data collected on the participants reflected the entangled complexity of their psychological, physical, and material experiences, rather than just their age and sex, as is currently typically the case.

As discussed above, there are physical characteristics of participants that are specifically relevant to sex/gender NI research such as head size (Barnes et al., 2010), given its relationship to brain volume. Similarly, height and weight should be noted in order to carry out the appropriate adjustments to brain volume measures; failure to do this must undermine the validity of any reports of sex differences in brain structure, as acknowledged by Ruigrok et al. (2014). There is the possibility that variations in hormone levels might produce (or mask) relevant sex/gender differences in brain structure and function. There is not currently strong evidence for such effects, but future research should be sure to take into account a range of sources of variation (e.g., diurnal, seasonal, and activity-related), and investigate variations in all research participants, as opposed to a singular focus, for example, on menstrual cyclicity and variations in women only. If there is a focus on hormonal variables, it should be noted that menstrual cycle phase is not, in fact, a good proxy for hormone fluctuations and direct measures will be required (Schwartz et al., 2012). Researchers should also be aware that popular beliefs/well-publicized claims regarding the psychological effects of menstrual phase on mood and male attractiveness ratings, have not been supported by recent meta-analyses (Romans et al., 2012; Wood et al., 2014, for contrary conclusion, see Gildersleeve et al., 2014, for critique, see Wood and Carden, in press).

If the basis of the research question is a link between measured differences in brain structure or activation patterns and behavioral or cognitive profiles, then a study’s dependent variables should obviously include appropriate measures of the relevant behavior or cognitive skill, and not just assume that such differences are well-known (and therefore do not need measuring) (Tomasi and Volkow, 2012). Whatever behavioral measures researchers choose in order to investigate the phenomenon of interest, it will be necessary to acknowledge that no sex/gender differences are “fixed” but contingent, the implication being that research findings will at best be a snapshot of the relationship of interest. Thus, an important research possibility is to additionally draw on the principles of contingency and entanglement to challenge the *stabilities* of observed differences and similarities, by experimenting with context or population, for example. This can be seen, for example, in studies investigating the extent to which training can alter pre-existing sex/gender differences in visuo-spatial processing (Feng et al., 2007). This type of research design would enable researchers to perform a “sensitivity analysis” of the conditions under which sex/gender is related to some kind of neural function or structure, facilitating knowledge of the stability and contingency of observed group differences. Hyde (2014) has similarly recommended a focus on the exploration of contexts in which gender differences appear and disappear as a way forward in such research.

#### Research models and hypotheses

Although whole brain analysis is possible with all NI techniques, many researchers choose to specify Regions of Interest (ROIs), particular areas of the brain identified as of interest due to previous research findings or predictions from particular neurocognitive models. This approach can, for example, reduce the multiple comparison problem resulting from comparing voxels across the whole brain. Where an ROI approach is chosen for either structure or function measures, the regions need to be clearly specified in advance (Poldrack et al., 2008) which may be difficult in the absence of a well-specified neurocognitive model (see Bluhm, 2013a). Researchers may instead be drawn to a priori hypotheses based on gender stereotypes (see Bluhm, 2013b), but clearly it needs to be carefully established whether such stereotypes are more than trivially true.

Changing models of brain–behavior relationships require adaptation of research exploring such relationships with attention to more and/or different categories of independent variables, including ways of capturing the role of the environment. McCarthy and Arnold (2011, p. 681) note the need for a “more nuanced portrayal of the types of variables that cause sex differences”, acknowledging that environmental influences “have an enormous effect on gender in humans and are arguably more potent in sculpting the gender-based social phenotype of humans” (p. 682). Jordan-Young (2010) and Jordan-Young and Rumiati (2012) similarly identify problems associated with the hard-wiring, “brain organization” theory in brain and brain development research and note that if researchers wish to bring understanding of how differences arise, then there is a need to focus more on the *dynamic* aspects of brain development, on the plasticity of the brain, and on identifying those events that enhance or change the course of development. For example, Cheslack-Postava and Jordan-Young (2012) reviewed research on the epidemiology of autism, focusing on studies that described or advanced explanations for the observed male preponderance in autism diagnosis. They found no studies that explored potential biosocial interactions of sex-linked biology and gender relations. Instead, the female/male difference was attributed to biological factors by default, though multiple lines of evidence suggest that gender could play a role in either the development of the disorder, or the likelihood of diagnosis once it is developed.

Given the major role played by NI itself in transforming our understanding of brain plasticity, it is surprising that there are so few examples of study design, cohort selection, and/or data interpretation where the entanglement of sex and gender is considered. The predominant approach is a “snapshot” comparison of females and males, which will only give limited insights regarding why, when or in whom such differences exist (Schmitz, 2002; Fine, 2013a,b). Importantly, although neuroscientists are well-aware that “in the brain” does not mean “hardwired”, the predominant use of “snap-shot” comparisons in sex/gender NI is guaranteed not to produce data that might challenge the idea of universal, fixed female/male brain differences (Fine, 2013a). The limitations of a “snapshot” approach should be acknowledged in the research design, where the choice of participants and/or their demographics should reflect more than just their biological sex (and possibly age) but also perhaps factors such as educational history and socio-economic and occupational status, with these factors controlled for in any subsequent analyses. Particular attention should be paid to the fact that there will be missing information concerning gendered socialization of participants. It is very probable that attitudes and behaviors of an individual have been sex-typically reinforced by the environment throughout her/his life and that development has been influenced by the particular importance of social learning in humans in combination with culturally shared gender stereotypes, norms, and roles (see Wood and Eagly, 2013). As identified above, assessment tools for measuring information about individual gender socialization are rare (Blakemore and Hill, 2008), no doubt in part because the whole process of gender socialization is highly complex and long-lasting, but also because it is mostly implicit and habitual, rather than deliberate. However, measures of gendered personal traits, attitudes, or cognitive development can indirectly reflect the effects of gender socialization.

Fine and Fidler (2014) have argued that the principles of overlap and mosaicism, together with the complexities arising from the consequences of contingency and entanglement, raise the important conceptual question of whether it makes sense at all to try to identify an effect size of the impact of biological sex on brain structure or function. But whatever precise research question is pursued, uncovering what are undoubtedly highly complex interactions against a background of noise and considerable individual differences will require more complex experimental designs. As the complexity of design increases, with multiple groups and multiple comparisons, so too must the sample size increase if adequate statistical power is to be achieved.

### Data analysis

Given the overlapping nature of sex/gender differences, it is important that effect sizes for each of the individual variables are reported. When studies reporting sex/gender differences only provide information about statistical differences, a misleading impression can be given of a near distinctive—or even oppositional—dichotomous finding. This was recently well illustrated by a large-scale (*n* = 949) report of significant sex differences in the structural connectome of the human brain (Ingalhalikar et al., 2014), accompanied by statements that the results “establish that male brains are optimized for intrahemispheric and female brains for interhemispheric communication” (p. 823). This was suggested to underpin “pronounced [behavioral] sex differences” (p. 826). However, no corrections for brain volume were made, and the actual effect sizes for brain differences were unreported, while behavioral differences in the larger population from which the sample were drawn were very modest (Joel and Tarrasch, 2014), being between 0 and 0.33 for behavioral differences, with 11 of 26 effect sizes being null/*d* < 0.1 (Gur et al., 2012).

A second statistical issue relating to the presentation of findings is the problematic statistical practice observed in neuroscience generally (Nieuwenhuis et al., 2011), as well as in NI sex/gender research (Kaiser et al., 2009; Bluhm, 2013a), of analyzing group data separately and then doing a “qualitative” comparison. Thus in sex/gender research, if a difference is found in one group and not the other, it is reported as a sex difference, even though no statistically significant difference has been established. In some cases, both within-group and direct comparisons are carried out, but only the former reported on. As Bluhm (2013a) points out, only by a direct statistical comparison, can a genuine difference be established, which should be illustrated by a single image showing the group differences, not 2 separate images for the 2 groups.

As will by now be clear, sex/gender NI research will require complex statistical frameworks to integrate the key variables associated with the participant cohort, to deal with the presence of potential nuisance variables, as well as incorporating imaging and behavioral data. This is obviously true of all NI research, and currently generally addressed by the use of General Linear Models (GLMs). However, the particularly “entangled” nature of the demographic, biological, and psychological variables in sex/gender research and the associated non-parametric nature of much of the data should be acknowledged if using a standard GLM analysis (Poline and Brett, 2012)—or, better, non-parametric methods such as permutation tests could be applied (Winkler et al., 2014). It is important that, whatever it comprises, the analysis pipeline is clearly specified (Bennett and Miller, 2010; Carp, 2012).

### Interpretation

The principle of overlap in gendered behavior is particularly important to bear in mind when it comes to inferring functional interpretations from neural differences (Fine, 2010b; Roy, 2012). It would seem obvious to add that this should be particularly true where there is no actual measure of the behavior/cognitive skill. The problematic nature of “reverse inference” is, of course, well-known in the neuroscientific community (e.g., Poldrack, 2006). In reverse inference, activation in particular brain regions is taken to equate to a specific mental process and, by extension, differences in activation can be taken to indicate differences in ability or efficiency. The danger is that gender stereotypes are inappropriately drawn upon in making such reverse inferences. This can happen particularly readily when, as is very often the case, there is no a priori neurocognitive model guiding hypotheses (Bluhm, 2013a; Fine, 2013a). This can lead to “stereotype-inspired” reverse inferences even where these are contradicted by behavioral similarity (see Fine, 2013a). In making reverse inferences that are consistent with gender stereotypes, different groups of researchers may even make precisely opposite assumptions about the behavioral significance of more vs. less activation in the same brain region (Bluhm, 2013b).

Although reverse inference is a generic issue in NI research, the ease and intuitive plausibility of such inferences in sex/gender NI studies makes it of particular concern. Reverse inference can certainly be a useful research tool when used to generate hypotheses to put to test in further work (Poldrack, 2008), and Fine (2013b) has noted the legitimacy of such an approach as part of a strategy of systematic development and testing of neurocognitive models and predictions. However, what is more common is to draw on stereotypical (and often inaccurate) assumptions about female/male differences in behavior or skill set *post hoc* to inform these inferences (Fine, 2013a). Given the sex/gender principle of overlap, this is poor scientific practice.

A final point of interpretation relates to entanglement. A recent review of sex/gender differences in decision-making “noted that we will use sex differences rather than gender differences in this review as we are focussed on biologically founded rather than culturally or socio-economically founded differences” (Van de Bos et al., 2013, p. 96). However, it is the nature of the entanglement problem that the variables of sex and gender are irreducibly entwined—it is not, in practice, possible to “control” for the gendered environment and examine only sex. This should be acknowledged, then, in the interpretation of findings. In addition, any evidence that the dependent variables being measured may be subject to alteration by training or focussed intervention should also be recognized. In addition, researchers should avoid framing findings of female-male differences as being “biological” or “fundamental”. Likewise, it is generally advisable to avoid the language that some variable is “affected by sex”, because that suggests the effect of biology apart from the gendered environment. Instead, the language “affected by sex/gender” or “linked with sex” would be preferable. It should, indeed, be considered that a study that approaches sex/gender as subject variable is only an ex-post facto study and, thus, it cannot demonstrate that sex/gender *causes* differences in any behavior (Brannon, 2008).

### Discipline-wide implications

While the aim of the recommendations above is to inform the planning, interpreting, and quality assessment of sex/gender research, we also think it is worth relating these issues to ongoing discussions regarding collective, discipline-wide strategies that could be helpful for ameliorating some of the issues in NI sex/gender research. One interesting proposal to consider is that of the “pre-registration” of protocols. This “in principle acceptance” (IPA) has recently been suggested in psychology circles (Chambers, 2013). A study protocol is submitted for peer review *before* the study is carried out; details include the relevant background literature and hypotheses, together with the specific procedures and analysis protocol (including sample size and a priori power analysis). Once accepted, the study is carried out exactly according to the agreed procedures and all findings published. This process could overcome many of those factors we have identified in this paper as significantly detrimental to NI sex/gender research. Publication bias could be reduced, as manuscript acceptance would be a function of the significance of the research question and associated methodology, not whether or not the results exceeded the magical *p* < 0.05 threshold. Thus, over time, it would be possible to better ascertain the ratio of negative to positive findings in any research sphere. While we acknowledge the value and role of exploratory research in the scientific research, declaring the analysis pipeline in advance would put constraints on practices such as *post hoc* data mining (Wagenmakers et al., 2011) and ensure that any failures to support hypotheses were identified as well as the converse. It could also preclude the *post hoc* introduction of interpretations, e.g., stereotypical assumptions about participant characteristics that were not measured as part of the study.

A long-standing proposal also relevant to some of the issues discussed here (see Fine and Fidler, 2014) is that of following the discipline of medicine in shifting away from null hypothesis significance testing towards an estimation approach of effect sizes and measures of their associated uncertainty, and greater use of meta-analysis. Proponents of the estimation approach (for extensive reviews, see Kline, 2004; Cumming, 2012), argue for a number of advantages over a null hypothesis significance testing approach, including reduced scope for false positive and false negative errors, and diminished conflation of statistical significance with practical or theoretical significance.

While the case for these two proposals is being made for behavioral science as a whole, the next two suggestions are more specific to sex/gender research, and arise out of the ease of default testing for sex/gender differences *post hoc*. One consequence of this is that the domain-general publication bias towards positive findings in behavioral science (Simmons et al., 2011; Fanelli, 2012; Yong, 2012) is greatly exacerbated in sex/gender research (e.g., Maccoby and Jacklin, 1974). Reviews of sex/gender NI research have demonstrated that this is a field that is indeed vulnerable to an overemphasis on positive findings and “loss” of null results (Bishop and Wahlsten, 1997; Fausto-Sterling, 2000; Kaiser et al., 2007; Fine, 2013a; see Figure 2). The first proposal is for the institutionalization of sex/gender similarity as well as difference in databases, to make it more likely that null findings are both recorded and identifiable. The second proposal is for editors of NI journals to request that sex/gender differences are replicated in an independent sample (obviously with discretion, depending on the rigor of the initial findings), to reduce the littering of the scientific literature with false-positive results.

Although, de facto, all research areas will wish to follow best practice guidelines, it is particularly important that the sex/gender NI research community is aware of the potential social significance of their findings (Roy, 2012; Schmitz, 2012). As reviewed elsewhere (e.g., Fine, 2012; Fine and Fidler, 2014), Choudhury et al. have argued that the representation of “brain facts” in the media, policy, and lay perceptions influence society in ways that can affect the very mental phenomena under investigation (Choudhury et al., 2009). This is illustrated in the upper part of Figure 1, whereby the result of the experiment itself, through its popularization, becomes part of gender socialization, and thus the experiment becomes entangled with the phenomenon of interest. With respect to NI research, this feedback effect may be enhanced by the popular and powerful impact of “brain facts” (Weisberg et al., 2008). The original finding of persuasive power of brain images (McCabe and Castel, 2008) has been disputed both qualitatively (Farah and Hook, 2013) and quantitatively in a recent meta-analysis (Michael et al., 2013). However, “brain facts”, regardless of the presence or absence of brain images, may enhance how satisfactory or valuable lay people judge scientific explanations of psychological phenomena to be (Morton et al., 2006; Weisberg et al., 2008; Michael et al., 2013). Gender essentialist thinking has been associated with a range of negative psychological consequences, including greater endorsement of gender stereotypes both in relation to self (Coleman and Hong, 2008) and others (Martin and Parker, 1995; Brescoll and LaFrance, 2004), stereotype threat effects (Dar-Nimrod and Heine, 2006; Thoman et al., 2008), greater acceptance of sexism, and increased tolerance for the status quo (Morton et al., 2009). This is in line with what Hacking (1995, p. 351) has described as “looping” or “feedback effects in cognition and culture”, whereby the causal understanding of a particular group changes the very character of the group, leading to further change in causal understanding. In other words, the outputs of sex/gender NI can affect the very object of their investigation, putting a particular responsibility on scientists to follow good practice guidelines for research. By taking steps to avoid false positives, to avoid the use of stereotypical reverse inferences, to give equal weight to sex/gender similarities as well as differences and to acknowledge the dynamic and entangled aspect of sex/gender variables, with research findings only representing a static “snapshot” in time, scientists can do much to avoid the undesirable consequences outlined above (see also Fine et al., 2013).

## Conclusion

**Figure 3:**
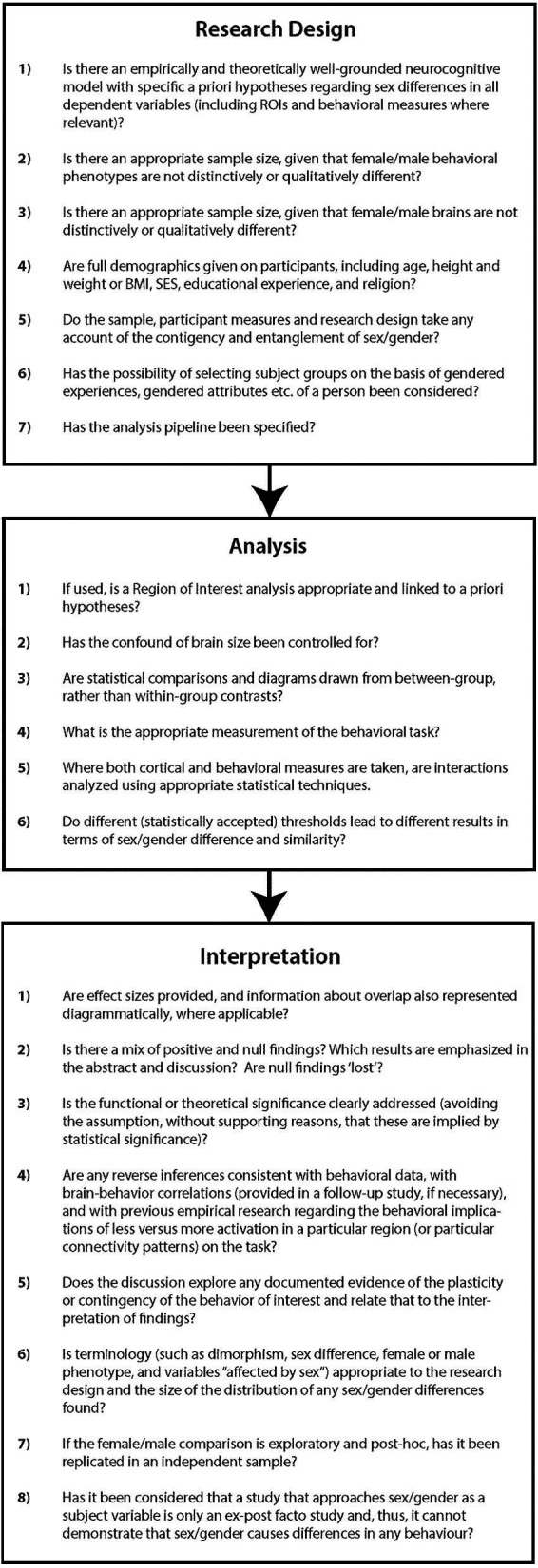
**Proposed guidelines for sex/gender research in neuroscience: critical questions for research design, analysis, and interpretation**.

We have outlined above the consequences for NI sex/gender research design, analytical protocols, and data interpretation of the four key principles of overlap, mosaicism, contingency, and entanglement and have summarized the consequences of these as a set of guidelines. These key principles and recommendations could also inform editorial boards and journal reviewers, as well as those who view, communicate, and interpret such research. In Figure 3, we offer a set of guidelines for the assessment of NI sex/gender research in order to assure that such research has addressed these implications (or, indeed, can). NI research is costly, time-consuming, and labor intensive. If it is to be applied in the field of sex/gender research then attention to the issues discussed here could reduce the incidence of under-informed research designs with consequent lack of reliable findings and/or waste of potentially valuable datasets. Changes to current research practices should result in a greater contribution to an understanding of the interaction between the neurobiology of the individual and the environment in which s/he develops and functions.

## Conflict of interest statement

The authors declare that the research was conducted in the absence of any commercial or financial relationships that could be construed as a potential conflict of interest.

## Footnotes

^1^As we describe below, neural phenotypes represent the complex entanglement of biological and environmental factors, such that it is generally not possible to entirely isolate the two. Thus, we use the composite term “sex/gender” as a way to refer to this irreducible complexity (see also Kaiser, 2012).

^2^Our focus in this paper is on the use of Magnetic Resonance Imaging MRI techniques, both structural and functional (fMRI). The majority of studies in this area, particularly those most commonly cited in the public domain, use MRI/fMRI techniques. We are aware that techniques with better temporal resolution such as electroencephalography (EEG) and magnetoencephalography (MEG) have been used in this field (and may, indeed, be more appropriate for the cognitive processes being investigated) but detailed inclusion of these is beyond the scope of this review. Almost all of the identified implications and recommendations will also be relevant to EEG and MEG research.

^3^By convention, a positive effect size refers to greater average male score, while a negative effect size refers to a greater average female score.

^4^The terms “female” and “male” here do not indicate an “innate” or “natural” neural maleness or femaleness, but are rather place-holders for a statistical approach that involves calculating the effect size of sex for a particular brain-related data set.
